# Antioxidant Activity and Anti-Inflammatory Effect of Blood Orange By-Products in Treated HT-29 and Caco-2 Colorectal Cancer Cell Lines

**DOI:** 10.3390/antiox14030356

**Published:** 2025-03-18

**Authors:** Rosa Calvello, Giusy Rita Caponio, Antonia Cianciulli, Chiara Porro, Melania Ruggiero, Giuseppe Celano, Maria De Angelis, Maria Antonietta Panaro

**Affiliations:** 1 Department of Biosciences, Biotechnologies and Environment, University of Bari Aldo Moro, Via Orabona 125, 70125 Bari, Italy; rosa.calvello@uniba.it (R.C.); giusy.caponio@uniba.it (G.R.C.); antonia.cianciulli@uniba.it (A.C.); melania.ruggiero@uniba.it (M.R.); 2 Department of Clinical and Experimental Medicine, University of Foggia, Via A. Gramsci 89/91, 71121 Foggia, Italy; chiara.porro@unifg.it; 3 Department of the Soil, Plant and Food Sciences (DiSSPA), University of Bari Aldo Moro, Via Amendola, 165/a, 70126 Bari, Italy; giuseppe.celano@uniba.it (G.C.); maria.deangelis@uniba.it (M.D.A.)

**Keywords:** intestinal inflammation, blood orange peel flour, by-products, TLR4, inflammasome, cytokines, oxidative stress

## Abstract

Blood orange peel flour (BO-pf)—a by-product of the citrus supply chain—still contains bioactive molecules with known health benefits, such as antiradical scavenging activity or an antiproliferative activity regarding tumors. In vitro studies have demonstrated that orange polyphenols showed potential involvement in necroptosis. In addition to previous research, we tested BO-pf on two colorectal cancer cell lines. Using HT29 and Caco2 cells, our experiments confirmed the regulation of inflammasome expression. They provided valuable insights into how BO-pf influences the cancer cell features (i.e., viability, proliferation, and pro- and anti-inflammatory activity). Notably, BO-pf extract is a rich source of polyphenolic compounds with antioxidant properties. Western blot and real-time PCR analyses showed that treatment with BO-pf extract demonstrated beneficial effects by influencing the expression of both pro-inflammatory cytokines (IL-1β, IL-6) through the modulation of the TLR4/NF-kB/NLRP3 inflammasome signaling. Moreover, the results of this study demonstrate that BO-pf extracts can enhance the expression of anti-inflammatory cytokines, such as IL-10 and TGFβ, suggesting that BO-pf extracts may represent a promising functional ingredient to counteract the intestinal inflammatory responses involved in IBD.

## 1. Introduction

Inflammatory bowel diseases (IBDs) are chronic inflammatory conditions of the gastrointestinal tract characterized by recurrent episodes of inflammation that dramatically impact human health. The two main types of IBD are Crohn’s disease (CD) and ulcerative colitis (UC) [1]. CD can affect any part of the gastrointestinal tract and is characterized by inflammation that can extend deep into the intestinal wall. At the same time, UC is limited to the colon and is characterized by continuous inflammation of the inner lining [2]. IBDs primarily affect young adults worldwide, and their incidence is also increasing among children [3].

IBDs are assumed to be produced by an unregulated response of intestinal immune and non-immune cells of the genetically predisposed host against the normal enteric microbiota, leading to mucosal destruction. However, the precise etiology of IBDs remains enigmatic despite intensive investigation.

Common symptoms of IBDs include diarrhea, abdominal pain, weight loss, and fatigue. In some cases, individuals may experience more severe symptoms, such as bloody stools and joint pain [4,5]. The intestinal mucosa represents a vital entry route for pathogenic bacteria, since enterocytes are the initial attack points for invasive microbes. The invasion of these entero-invasive microorganisms activates innate immune cells, leading to excessive production of pro-inflammatory mediators such as cytokines and chemokines, which, in turn, recruit other immune cells of the intestinal wall, inducing damage to the enteric epithelium with loss of barrier function and causing a further amplification of the inflammatory response [6]. Moreover, the impaired intestinal epithelial barrier, a hallmark of IBDs, is also influenced by oxidative stress. Reactive oxygen species (ROS) can damage tight junctions between epithelial cells, increasing permeability and allowing the passage of luminal antigens and bacteria. This can trigger an altered intestinal inflammatory and immune response, exacerbating and perpetuating the inflammatory process. Therefore, suppressing this hyperactive intestinal inflammatory process could represent a promising therapeutic strategy. Fruit peel extracts of several citrus species, such as the orange, are a valuable source of bioactive compounds, including polyphenols, flavonoids, phenolic acids, and carotenoids, which have been extensively studied for their antioxidant and anti-inflammatory properties [7,8]. These compounds have demonstrated the ability to neutralize harmful free radicals, reducing oxidative stress and protecting cells from damage [9,10,11].

Previously, an orange-enriched diet has been shown to improve the fecal microbiome and metabolome in metabolic dysfunction-associated fatty liver disease patients [12]. The microbial metabolic pathway is affected by diet and is correlated with intestinal permeability in various diseases [13]. Previous research has also highlighted the potential therapeutic applications of polyphenols in multiple diseases. In particular, their antioxidant properties have shown promise in mitigating the risk of cardiovascular disease by reducing inflammation, improving blood lipid profiles, and protecting against atherosclerotic plaque formation [11,14].

Given their abundance of bioactive compounds and demonstrated health benefits, orange peel extracts represent a promising natural source of therapeutic agents for various conditions [15].

Because of the growing evidence of anti-inflammatory bioactivity in fruit extracts, we hypothesized that the antioxidant properties of blood orange peel flour (BO-pf) extracts could mitigate the inflammatory response and protect the intestinal epithelial barrier in IBDs. Thus, our study aimed to investigate the protective effects of BO-pf extracts in two human colon tumor cell lines, HT29 and Caco2, submitted to Lipopolysaccharide (LPS) treatment, a component of the cell wall of Gram-negative bacteria that induces a strong inflammatory response through interaction with several microbial product receptors. These tumor cell lines are widely used in in vitro models for studying the structural and functional features of normal human differentiated intestinal epithelial cells [16,17]. Specifically, BO-pf extract has previously been characterized for antioxidant activity and total phenol content. Moreover, UHPLC-DAD analysis was performed to determine the concentration of the primary polyphenols in the extract. Thus, we tested the effects of different concentrations of BO-pf on cancer cell features (i.e., cell viability, proliferation, and inflammation) in HT29 and Caco2 cells.

## 2. Materials and Methods

### 2.1. Chemical Characterization of Blood Orange Peel Flour (BO-pf)

#### 2.1.1. BO-pf Extract Preparation

Blood orange peel flour (BO-pf), supplied by the company Packtin Srl (Reggio nell’Emilia, Italy), was obtained through cold drying of the orange pulp without the addition of additives, colorings, or preservatives. The BO-pf extract was prepared following the methods described by Caponio et al., with some modifications [18]. Briefly, 3 g of BO-pf was mixed with water (1:10 *w*/*v*), vortexed for 10 min, sonicated for 15 min (Elmasonic S 60 H, ELMA, Singen, Germany), and then centrifugated at 12,000× *g* for 10 min (SL 16R Centrifuge, Thermo Fisher Scientific, Waltham, MA, USA) to obtain the extract. The extraction process was repeated twice more with 30 mL of water. The three extracts were combined, filtered as previously described, and stored at −20 °C until analysis. All extracts were prepared in triplicate.

#### 2.1.2. Total Phenolic Content and Antioxidant Activity

The BO-pf extract’s total phenolic content (TPC) was determined using the Folin–Ciocalteu method, as described by Caponio et al. [19]. Briefly, 20 μL of the appropriately diluted extract was added to 980 μL of Milli-Q water, followed by 100 μL of Folin–Ciocalteu reagent. After 3 min, 800 μL of a 7.5% Na_2_CO_3_ solution was added, and the mixture was then incubated in the dark for 60 min. Absorbance was measured at 720 nm using an Evolution 60 s UV–visible spectrophotometer (Thermo Fisher Scientific, Rodano, Italy). Results were expressed as milligrams of gallic acid equivalents (GAE) per gram of dry weight (DW) sample (mg GAE/g DW). Each sample was analyzed in triplicate. The antioxidant activities of the extracts were assessed using ABTS and DPPH assays, following the method reported by Caponio et al. [19]. The ABTS [2,2′-azino-bis(3-ethylbenzothiazoline-6-sulfonic acid)] radical was produced through a reaction with potassium persulfate (K_2_S_2_O_8_). Specifically, 25 mL of ABTS (7 mM in water) was combined with 440 μL of K_2_S_2_O_8_ (140 mM) and left in the dark at room temperature for 12–16 h. The working solution was then diluted with water to achieve a final absorbance of 0.80 ± 0.02 at 734 nm [20]. The reduction in absorbance was recorded at 734 nm after 8 min of incubation, and results were expressed as μmol of Trolox equivalents (TE) per gram of DW. For the DPPH (2,2-diphenyl-1-picrylhydrazyl) assay, a 0.08 mM DPPH solution was prepared in ethanol. In spectrophotometric cuvettes, 50 μL of each sample was mixed with 950 μL of the DPPH solution. After incubating in the dark for 30 min, the decrease in absorbance was measured at 517 nm. Each sample was analyzed in triplicate.

#### 2.1.3. Determination of DPPH Free Radical Scavenging Activity

The method described by Nićiforović et al. [21] was used with some modifications. Briefly, DPPH (8 mg) was dissolved in EtOH (100 mL) to obtain a concentration of 80 μg/mL. Serial dilutions were carried out with the stock solutions (1 mg/mL) of the BO-pf extract. Solutions (500 µL each) were mixed with DPPH (500 µL) and allowed to stand for 30 min for any reaction, and the absorbance was measured at 517 nm. A control sample was prepared containing the same volume without BO-pf extract. For the blank, 95% methanol was used. The DPPH free radical scavenging activity (%) was calculated using the following equation:

$$\%inhibition=\left(\frac{Ac-As}{Ac}\right)\times 100$$

where Ac is the absorbance of the control, and As is the absorbance of the sample.

The IC50 value, which is the concentration of the BO-pf that reduces 50% of the free radical concentration, was calculated as μg/mL through a sigmoidal dose–response curve.

#### 2.1.4. Naringin and Neohesperidin Determination by UHPLC-DAD

A UHPLC Ultimate 3000RS Dionex system (Thermo Fisher Scientific, Waltham, MA, USA) was used to analyze naringin and neohesperidin. The UHPLC setup included a quaternary pump, an autosampler, a column compartment, and a detector. Analytical separation was carried out based on a previously reported method, with slight modifications [22]. A Hypersil GOLD aQ C18 column (100 mm long, 2.1 mm internal diameter, and 1.9 μm particle size) was utilized, maintained at 30 °C, with a constant flow rate of 0.3 mL min^−1^. The mobile phase consisted of water with formic acid (90:10 *v*/*v*) as solvent A and acetonitrile with formic acid (99.9:0.1 *v*/*v*) as solvent B. The gradient program for solvent A was as follows: 0–26 min, decreasing from 94% to 45%; 26–33 min, further reducing to 30%; 33–35 min, held isocratically at 30%. The system was then re-equilibrated to its initial conditions for 9 min. The PDA detector was set to scan from 220 to 600 nm of wavelength managed by a 3D field. Quantitative analysis was performed according to the external standard method based on calibration curves obtained by injecting different concentrations of standard solutions of naringin and neohesperidin (Sigma-Aldrich, St. Louis, MO, USA).

#### 2.1.5. Proximate Composition

The moisture content was measured using a thermobalance (Ladwag MAC 110/NP, Radwag, Wagi Elektroniczne, Radom, Poland). Protein content (total nitrogen × 6.25), ash, lipid, and total dietary fiber were determined following AOAC methods 979.0, 923.03, 945.38, and 985.29, respectively [23]. Carbohydrate content was estimated by difference, subtracting the total dietary fiber, protein, ash, moisture, and lipid contents from 100. All analyses were performed in triplicate.

### 2.2. In Vitro Assays of BO-pf Samples on Cell Cultures

#### 2.2.1. Cell Cultures and Treatments

Human colorectal adenocarcinoma HT29 cells (ICLC HTL99026) and human colorectal cancer Caco2 cells (ICLC HTL97023) were both obtained from Interlab Cell Line Collection (Genoa, Italy) and cultured in D-MEM supplemented with L-glutamine (2 mM), 100 U/mL penicillin, 100 μg/mL of streptomycin, and 10% fetal bovine serum (FBS UE approved origin) (all reagents were purchased from Life Technologies-Invitrogen, Milan, Italy), henceforth referred to as complete medium. Both cell cultures were maintained at 37 °C in a humidified atmosphere containing 5% CO_2_ atmosphere and expanded in tissue culture flasks (75 cm^2^, BD Biosciences, Milan, Italy), with the medium changed every 2 days. The cells were seeded in six-well cell culture plates and 96-multiwell plates, cultured to reach 80% confluency, and then submitted to subsequent treatments.

For the experiments, cells were treated with 1 μg/mL *Salmonella enterica Typhimurium* LPS (Sigma-Aldrich, Milan, Italy) for 24 h, according to preliminary experiments. Before LPS stimulation, some wells were pre-treated with different concentrations of BO-pf extract (25, 30, 50, 100, 200, and 500 μg/mL). After one hour of incubation at 37 °C, cell cultures were stimulated with LPS as previously indicated. Untreated cells were used as control.

#### 2.2.2. Cell Viability Assay

To test cell viability, we used a (3,4,5-dimethylthiazol-2-yl)-2-5-diphenyltetrazolium bromide (MTT) assay, based on the reduction of MTT by the mitochondrial dehydrogenase to a purple formazan product in vital cells (Sigma-Aldrich) [24]. Briefly, cells (1 × 10^4^) were seeded in a 96-well plate (BD Biosciences). After cell treatment, culture media were carefully removed, and 100 μL of 0.5 mg/mL MTT in cell culture medium was added to each well. At the end of incubation for 4 h, 150 μL of Dimethylsulfoxide (DMSO) was added to each well for 20 min under stirring to dissolve the formed formazan crystals. Cell viability was measured by reading the absorbance (560 nm) on a Cytation 3 Cell Imaging Multi-Mode Reader (Biotek, Winooski, VT, USA). The cell viability was calculated according to the following formula: % cell viability = [OD (560 nm) tested compound/OD (560 nm) control cells] × 100. Values were expressed as the average percentage ± SD.

#### 2.2.3. Electrophoresis and Western Blotting

After in vitro treatments, cells were lysed for 30 min on ice with lysis buffer [1% Triton X-100, 20 mM Tris–HCl, 137 mM NaCl, 10% glycerol, two mM EDTA, one mM phenylmethylsulfonyl fluoride (PMSF), 20 μM leupeptin hemisulfate salt, 0.2 U/mL aprotinin (Sigma-Aldrich)]. The lysate, vortexed for 15–20 s, was centrifuged at 12,800× *g* for 20 min. The protein concentration in the supernatant was spectrophotometrically determined using Bradford’s protein assay. Briefly, protein samples were diluted with sample buffer (0.5 M Tris HCl pH 6.8, 10% glycerol, 10% *w*/*v* SDS, 5% β2-mercaptoethanol, 0.05% *w*/*v* bromophenol blue) and then boiled for 3 min. Proteins (25 μg/lane) and prestained standards (Bio-Rad Laboratories, Hercules, CA, USA) were loaded on 4–12% SDS precast polyacrylamide gels (Life Technologies, Milan, Italy) to be separated by SDS-PAGE.

After performing electrophoresis, proteins were then transferred to a nitrocellulose membrane, blocked with 5% (*w*/*v*) non-fat dried milk for 1 h, and then washed 3 times with 0.1% Tween 20-PBS (T-PBS). Then, membranes were incubated overnight at 4 °C with primary mouse monoclonal antibody (moAb) anti-β-actin (sc-47778), rabbit poAb anti-TLR4 (sc-10741), rabbit poAb anti-Caspase 1 (sc-622), mouse moAb anti-p-IkBα (sc-8404) (all from Santa Cruz Biotechnology, Inc., Milan, Italy), and rabbit poAb anti-NLRP3 (ab-214185) (from Abcam, Cambridge, UK), all used at a 1:500 dilution. The membranes were washed with T-PBS (for 20 min, 3 times) and then incubated for 60 min at room temperature on a shaker with a secondary antibody (1:10,000, Bentham, Milan, Italy), anti-mouse or anti-rabbit IgG conjugated to horseradish peroxidase (HRP). After three washes with 0.1% T-PBS, immunoreactive bands were acquired using a ChemiDoc XRS + Imager (Bio-Rad Laboratories, Hercules, CA, USA). The optical density of each band was normalized to the corresponding β-actin level and expressed as mean ± SD. Bands were visualized using the chemiluminescence method (Bio-Rad Laboratories, Hercules, CA, USA).

#### 2.2.4. Reverse Transcriptase-Polymerase Chain Reaction (RT-PCR) and Quantitative Real-Time PCR Analyses

Total cell RNA was extracted by using a GenElute™ Mammalian Total RNA Miniprep Kit (Sigma-Aldrich) according to the manufacturer’s instructions. Then, RNA was reverse transcribed back into cDNA with SuperScript III Reverse Transcriptase (Thermo Fisher Scientific, Milan, Italy), and the expression rates of the mRNA levels of various genes were quantified using the SYBR Green QuantiTect RT-PCR Kit (Roche, South San Francisco, CA, USA). β-actin was used as an endogenous reference. Data were analyzed using the relative standard curve method according to the manufacturer’s protocol. The mean value of each gene after β-actin normalization at the time point showing the highest expression was used as a calibrator to determine the relative levels. The primers used for amplification were IL-1β (XM_047444175.1) forward primer 5′-CACGATGCACCTGTACGATCA-3′, reverse primer 5′-GTTGCTCCATATCCTGTCCCT-3′; IL-6 (NM_000600.5) forward primer 5′-CTGGATTCAATGAGGACACTTGC-3′, reverse primer 5′-TCAAATCTGTTCTGGAAGGTACTCTAGG-3′; IL-10 (NM_000572.3) forward primer 5′-AGAACCTGAAGACCCTCAGGC-3′, reverse primer 5′-CCACGGCCTTGCTCTTGTT-3′; TGFβ (NM_000660.7) forward primer 5′-TGAACCGGCCTTTCCTGCTTCTCATG-3′, reverse primer 5′-GCGGAAGTCAATGTACAGCTGCCGC-3′; and, for β-actin (NM_001101.5), forward primer, 5′-GGCGGCACCACCATGTACCCT-3′, reverse primer, 5′-AGGGGCCGGACTCGTCATACT-3′. One microliter of cDNA was amplified in 25 μL of PCR solution (11.5 μL of cDNA solution in water, 1 μL of primer sets, and 12.5 μL of Power SYBR Green PCR Master Mix; Thermo Fisher) in a 7500 Real-time PCR System (Applied Biosystems, Monza, Italy), and fluorescence was monitored at each cycle. Cycle parameters were 95 °C for 15 min to activate Taq, followed by 40 cycles of 95 °C for 15 s, 55 °C for 1 min, and 72 °C for 1 min. Serial dilutions of cDNA from the same source as samples were used to obtain a standard curve. The individual targets for each sample were quantified by determining the cycle threshold and by comparison with the standard curve. The relative amount of the target mRNA was normalized to the level of β actin mRNA.

### 2.3. Statistical Analysis

The data were presented as mean ± standard deviation (SD). Statistically significant differences (*p* ≤ 0.05) were evaluated using one-way analysis of variance (ANOVA) with a subsequent Tukey test for multiple comparisons. The studies used Minitab statistical software version 21.1.0 (Minitab Inc., State College, PA, USA).

## 3. Results

### 3.1. Chemical Characterization of BO-pf

In the current study, we established the anti-inflammatory function of BO-pf in cancer cell lines.

Before that, we deeply characterized BO-pf for its chemical composition (moisture, protein, lipid, carbohydrate, fiber, and salt), antioxidant activity, and phenolic content, as shown in Table 1. The results show that BO-pf is low in water, as confirmed by the moisture level of about 8.14 g/100 g, and rich in protein (5.08 g/100 g). Notably, BO-pf represents a source of fiber with a value content of 35.2 ± 0.08. However, this by-product contains a low fat content of about 1.12 g/100 g.

Moreover, the BO-pf extract was also tested for antioxidant activity by ASTS and DPPH assays. The results in Table 1 demonstrate a significant antioxidant activity, as shown by ABTS and DPPH assays of 21.94 ± 0.59 µmol TE/g and 20.87 ± 0.40 µmol TE/g, respectively. The IC_50_ of the aqueous extract of BO-pf was recorded with a value of 725 μg/mL. Furthermore, the total phenolic content (TPC) of BO-pf, measured by the Folin–Ciocalteu method, reached a value of 22.64 ± 0.20 mg GAE/g. Specifically, the main phenolic components detected in the samples and quantified by UHPL-DAD analysis were neohesperidin (1.96 ± 0.06 mg/g) and naringin (0.88 ± 0.06 mg/g).

### 3.2. Anti-Inflammatory Activity

#### 3.2.1. Effects of BO-pf Extract on the Intestinal Cells’ Viability

**Figure 1:**
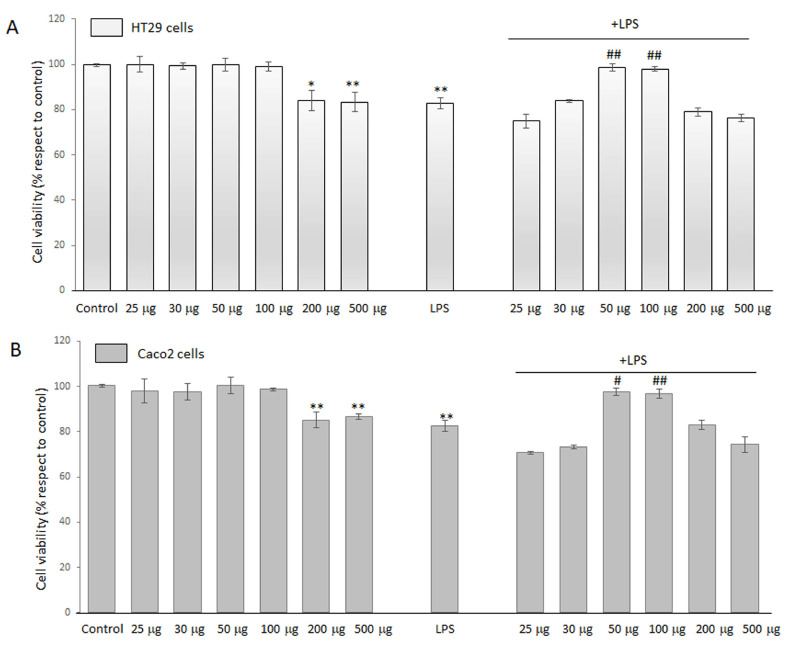
Cell viability analysis. HT29 (panel **A**) and Caco2 (panel **B**) cells were treated with different concentrations of BO-pf extract ranging from 25 to 500 µg/mL, alone or with LPS (1 µg/mL). Untreated cells represent the control. Cell viability was evaluated after 24 h by MTT assay. Data were expressed as means ± SD of five independent experiments. (** *p* < 0.01 and * *p* < 0.05 vs. Control; ## *p* < 0.01 and # *p* < 0.05 vs. LPS).

MTT assay was used to quantitatively evaluate cell viability and determine whether BO-pf extract caused toxicity in LPS-treated HT29 and Caco2 cells. Preliminarily, we evaluated the optimal BO-pf extract concentrations on HT29 and Caco2 cell lines. For this purpose, cells were treated with different concentrations of BO-pf extract, ranging from 25 to 500 µg/mL for 24 h. Data reported in Figure 1 show that extract concentrations of 200 µg/mL and 500 µg/mL determined a significant reduction in cell viability in both cell types compared to untreated cells. In contrast, BO-pf extract concentrations from 25 to 100 µg/mL did not exert any cytotoxic effect in HT29 (Figure 1, panel A) or Caco2 (Figure 1, panel B) cells. Next, to verify the combined effect on cell viability, BO-pf extract was tested in the presence of LPS (1 µg/mL). In this respect, both intestinal cell types were pre-treated with extract for one hour, and later, LPS was added. We observed that treatment for 24 h with LPS alone significantly reduced cell viability compared to control cells (*p* < 0.01). Interestingly, we have highlighted that the co-incubation of BO-pf extract at 50 and 100 µg/mL concentrations in the presence of LPS exhibited a cytoprotective effect against LPS-induced damage in both cell lines. Therefore, the BO-pf extract concentrations of 50 and 100 µg/mL were used in our experiments, as they effectively elicited an anti-inflammatory response in both HT29 and Caco2 cells.

#### 3.2.2. BO-pf Extract Treatment Effect on TLR4 Modulation in Intestinal Cells

**Figure 2:**
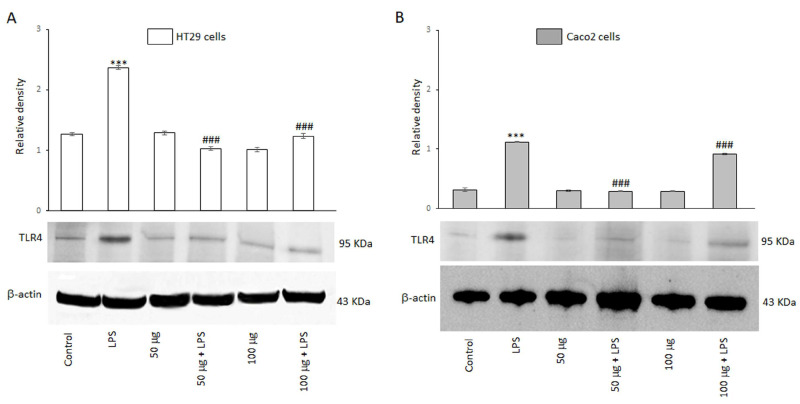
Effects of BO-pf extract on TLR4 expression levels. Western blotting TLR4 detection in HT29 (panel **A**) and Caco2 (panel **B**) untreated cells (Control), treated for 24 h with LPS (1 µg/mL) alone (LPS) or with LPS after pre-treatment of BO-pf extract (50 and 100 µg/mL). Densitometric analysis of TLR4 expression after normalization against β-actin is reported. Data are presented as means ± SD of five independent experiments. (*** *p* < 0.001 vs. Control, ### *p* < 0.001 vs. LPS).

Toll-like receptor 4 (TLR4) is a member of the TLR family and plays a key role in mediating inflammatory responses by recognizing bacterial endotoxins. The ability of the BO-pf extract to modulate the expression of the TLR4 receptor was evaluated by Western blot analysis. Figure 2 shows that HT29 and Caco2 cells express this receptor, as evidenced by a 95 kDa protein band corresponding to TLR4. The results also revealed that TLR4 protein levels were significantly (*p* < 0.001) increased in both cell lines following a 24 h treatment with LPS, compared to the control (Figure 2, panels A and B). However, the BO-pf extract co-incubation modulated the expression level of the TLR4 receptor, inducing a significant decrease (*p* < 0.001) at both 50 and 100 µg/mL concentrations of the extract in both cell lines compared to LPS alone. It should be emphasized that the 50 µg/mL concentration was more effective in significantly reducing the expression of TLR4 receptors in two cell lines. These findings suggest that BO-pf extract at the two concentrations used could downregulate the level of TLR4.

#### 3.2.3. Effects of BO-pf Extract on Pro-Inflammatory Mediator Expression in Intestinal Cells

**Figure 3:**
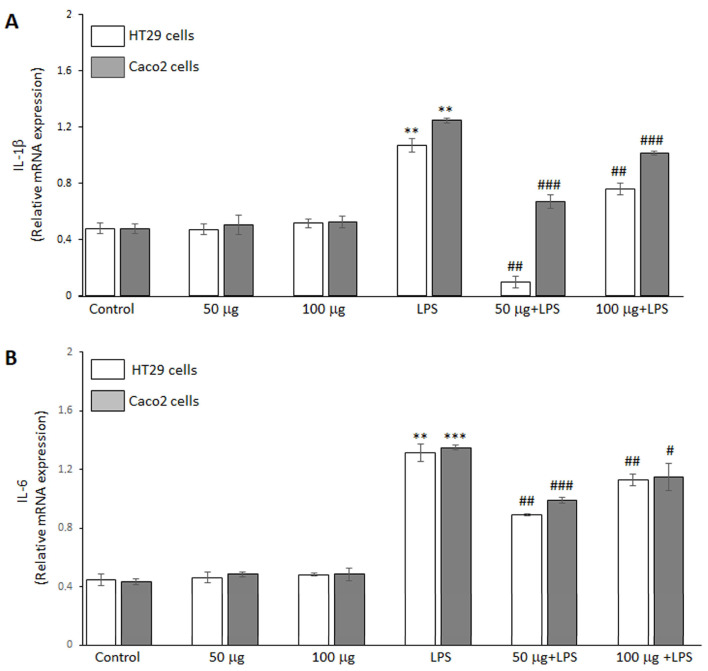
Analysis of pro-inflammatory cytokine expression. Real-time PCR analysis of IL-1β (panel **A**) and IL-6 (panel **B**) mRNA expression levels in HT29 and Caco2 cells treated with LPS (1 µg/mL) alone (LPS) or after pre-treatment with BO-pf extract (50 and 100 µg/mL). Untreated cells represent the control. Data represent the mRNA fold changes relative to β-actin used as resident control and expressed as means ± SD of five independent experiments. (*** *p* < 0.001 and ** *p* < 0.01 vs. Control; ### *p* < 0.001, ## *p* < 0.01, and # *p* < 0.05 vs. LPS).

The ability of the BO-pf extract to regulate the inflammatory response in HT29 and Caco2 cells was analyzed by RT-PCR analysis. The data demonstrated that LPS endotoxin significantly increased IL-1β (Figure 3, panel A) and IL-6 (Figure 3, panel B) cytokine expression compared to untreated cells in both intestinal cell lines. Furthermore, treatment with the BO-pf extract alone resulted in IL-1β and IL-6 levels comparable to those observed in control cells at both tested concentrations (Figure 3, panels A and B). Interestingly, in LPS-stimulated HT29 and Caco2 cells, pre-treatment for 1 h with BO-pf extract significantly downregulated the expression of these pro-inflammatory mediators at the two concentrations tested. Again, it should be noted that the 50 µg/mL concentration was more effective in reducing the expression of IL-1β and IL-6 in both cell lines. These results indicate that BO-pf extract attenuates the pro-inflammatory signaling at the transcript level.

#### 3.2.4. Effects of BO-pf Extract on Anti-Inflammatory Mediator Expression in Intestinal Cells

**Figure 4:**
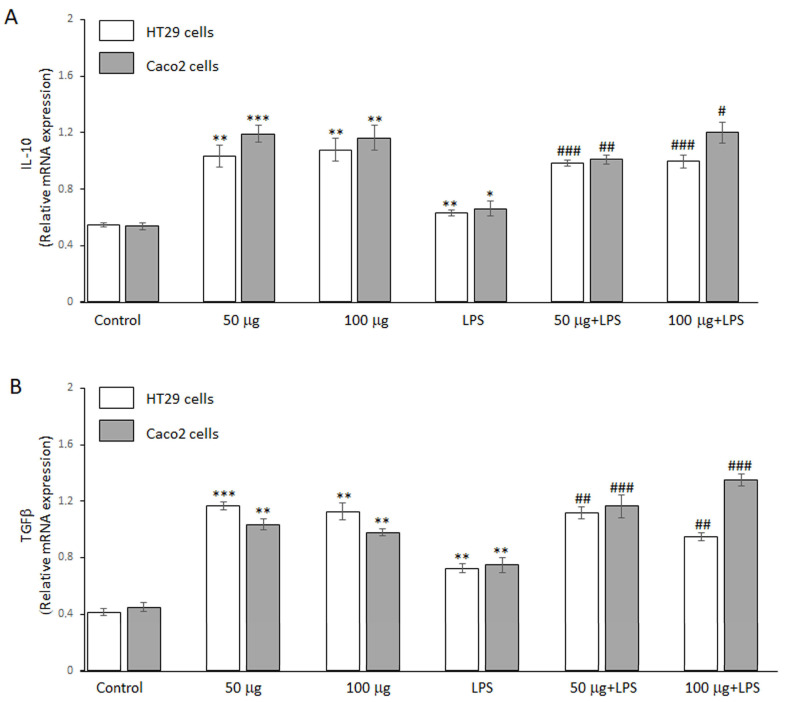
Analysis of anti-inflammatory cytokine expression. Real-time PCR analysis of IL-10 (panel **A**) and TGFβ (panel **B**) mRNA expression levels in HT29 and Caco2 untreated cells (Control), treated for 24 h with LPS (1 µg/mL) alone (LPS) or with LPS after pre-treatment with BO-pf extract (50 and 100 µg/mL). Data represent the mRNA fold changes relative to β-actin used as resident control and expressed as means ± SD of five independent experiments. (*** *p* < 0.001, ** *p* < 0.01 and * *p* < 0.05 vs. Control; ### *p* < 0.001, ## *p* < 0.01, and # *p* < 0.05 vs. LPS).

The potential anti-inflammatory effect of BO-pf extract was further evaluated by RT-PCR analysis. In this regard, we assessed its ability to regulate the anti-inflammatory genes IL-10 and TGFβ expression levels in HT29 and Caco2 cells. Figure 4 shows that, in the absence of LPS, the two concentrations of the BO-pf extract tested in both cells were able to significantly upregulate the expression levels of cytokines IL-10 (Figure 4, panel A) and TGFβ (Figure 4, panel B) compared to control cells. In addition, a significant increase in the expression of two anti-inflammatory cytokines was also observed in both intestinal cells treated with LPS alone compared to control cells. Pre-treatment with 50 and 100 μg/mL BO-pf extract for 1 h, followed by LPS stimulation for 24 h, resulted in further upregulation of IL-10 and TGFβ mRNA expression in both cell lines, compared to cells treated with LPS alone. These data suggest that the BO-pf extract ameliorates intestinal inflammatory responses at the transcriptional level.

#### 3.2.5. Effects of BO-pf Extract on NLRP3 Inflammasome Expression in Intestinal Cells

**Figure 5:**
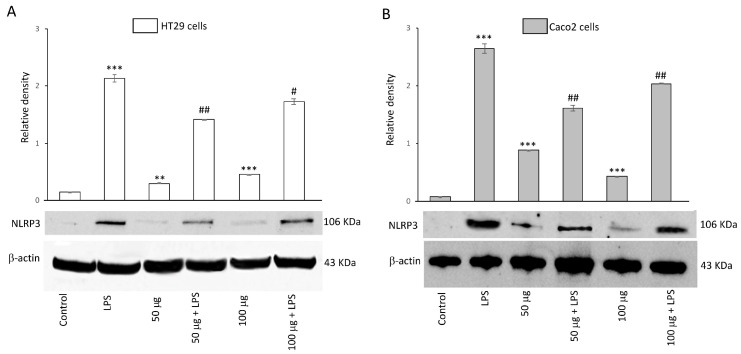
Effects of BO-pf extract on NLPR3 expression levels. Western blotting NLPR3 detection in the HT29 (panel **A**) and Caco2 (panel **B**) untreated cells (Control), treated for 24 h with LPS (1 µg/mL) alone (LPS) or with LPS after pre-treatment with BO-pf extract (50 and 100 µg/mL). Densitometric analysis of NLPR3 expression after normalization against β-actin is reported. Data are presented as means ± SD of five independent experiments. (*** *p* < 0.001 and ** *p* < 0.01 vs. Control; ## *p* < 0.01 and # *p* < 0.05 vs. LPS).

To evaluate the inhibitory capability of BO-pf extract on NLRP3 activation, HT29 and Caco2 cells were treated with 50 and 100 µg/mL BO-pf extract both in the presence and the absence of LPS. Both concentrations of BO-pf extract caused a slight but significant alteration in the level of NPLR3 compared to control cells in both cell types (Figure 5, panels A and B). Intestinal cells treated with LPS for 24 h showed a significant increase in the expression level of NLRP3 inflammasome compared to untreated cells. Interestingly, pre-treatment of cell cultures with BO-pf extract before LPS stimulation resulted in significant downregulation of NLRP3 expression at both concentrations tested, with a more pronounced decrease observed at the 50 µg/mL concentration in both intestinal cells. Overall, the results show that BO-pf extract downregulates the expression of NLRP3, an intracellular protein complex that triggers inflammatory responses in the intestine.

#### 3.2.6. Effects of BO-pf Extract on Caspase-1 Expression in Intestinal Cells

**Figure 6:**
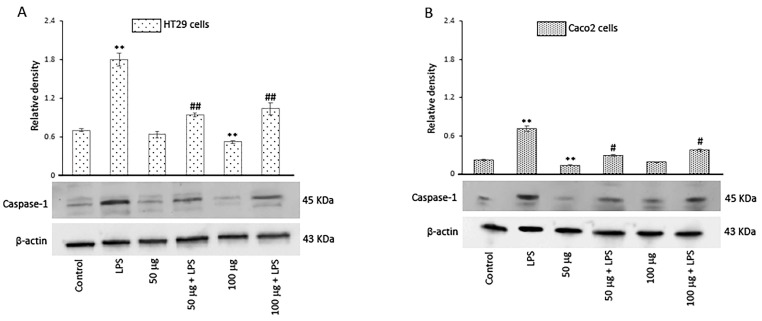
Effects of BO-pf extract on caspase-1 expression levels. Western blotting caspase-1 detection in the HT29 (panel **A**) and Caco2 (panel **B**) untreated cells (Control), treated for 24 h with LPS (1 µg/mL) alone (LPS) or with LPS after pre-treatment with BO-pf extract (50 and 100 µg/mL). Densitometric analysis of caspase-1 expression after normalization against β-actin is reported. Data are presented as means ± SD of five independent experiments. (** *p* < 0.01 vs. Control, ## *p* < 0.01 and # *p* < 0.05 vs. LPS).

The activation of inflammasomes, a group of intracellular multimeric protein complexes that activate inflammatory caspase-1, is a major inflammatory pathway. In this regard, we evaluated the effect of both concentrations of BO-pf extract on caspase-1 expression levels. Our results showed that, in LPS-treated cells, the caspase-1 expression significantly increased in comparison to control cells. Conversely, in both cell types, there was a significant reduction in the level of caspase-1 in LPS-activated cells pre-treated with BO-pf extract compared to cells treated with LPS (Figure 6, panels A and B).

#### 3.2.7. Effects of BO-pf Extract on NF-kB Signaling Pathway in Intestinal Cells

**Figure 7:**
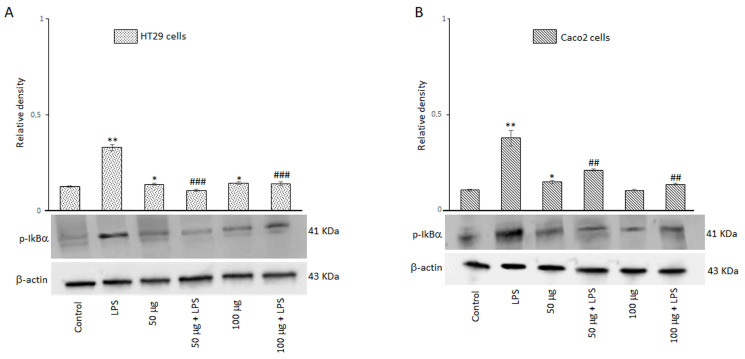
Effects of BO-pf extract on p-IkBα expression levels. Western blotting p-IkBα detection in the HT29 (panel **A**) and Caco2 (panel **B**) untreated cells (Control), treated for 24 h with LPS (1 µg/mL) alone (LPS) or with LPS after pre-treatment with BO-pf extract (50 and 100 µg/mL). Densitometric analysis of p-IkBα expression after normalization against β-actin is reported. Data are presented as means ± SD of five independent experiments. (** *p* < 0.01 and * *p* < 0.05 vs. Control, ### *p* < 0.001 and ## *p* < 0.01 vs. LPS).

A signal model for NLRP3 inflammasome activation is represented by a priming signal provided by microbial components or endogenous cytokines. This leads to the activation of the NF-kB through IkB phosphorylation on specific serine residues and subsequent upregulation of NLRP3. For this reason, we evaluated the effect of BO-pf extract on p-IkBα expression in HT29 and Caco2 cells treated with 50 and 100 µg/mL BO-pf extract both in the presence and the absence of LPS. As expected, intestinal cells treated with LPS for 24 h showed a significant increase in the expression level of p-IkBα compared to untreated cells. Interestingly, pre-treatment of cell cultures with BO-pf extract before LPS stimulation resulted in significant downregulation of p-IkBα expression at both concentrations tested (Figure 7, panels A and B). Overall, the results show that BO-pf extract, through p-IkBα expression modulation, downregulates the expression of NF-kB, an intracellular protein complex that triggers inflammatory responses in the intestine.

## 4. Discussion

In recent years, the agro-food industry has increasingly focused on valorizing by-products derived from food processing, aiming to reduce waste and promote circular economy and sustainability models. In this context, orange peel flour represents a virtuous example of reuse, as it is a by-product rich in bioactive compounds and nutrients that can be recovered and employed in various fields, from functional food to cosmetics and pharmaceuticals [25].

From a nutritional and functional perspective, BO-pf is distinguished by its high content of fiber, polyphenols, and flavonoids, including neohesperidin and naringin, which are known for their antioxidant and anti-inflammatory properties [26]. These bioactive compounds add value to the product and open new opportunities for its use as a functional ingredient in bakery products, nutritional bars, and supplements, thus contributing to the development of healthier and more sustainable foods. Specifically, the chemical composition of BO-pf, particularly its high fiber content (35.2 g/100 g) and moderate protein level (5.08 g/100 g), makes it comparable to plant-based by-products that have already been studied for their use in the formulation of functional foods or supplements [27]. Moreover, its low fat content (1.12 g/100 g) and reduced moisture (8.14 g/100 g) suggest more excellent stability and shelf-life, which are essential factors for its application in nutraceuticals [28,29]. The antioxidant activity of BO-pf aligns with previous studies on citrus by-products, where the abundance of phenolic compounds has been linked to high antioxidant potential [8,30,31]. The results of the DPPH scavenging activity of BO-pf, with an IC_50_ value of 725 μg/mL, demonstrate lower free radical scavenging activity compared to ascorbic acid and BHT, which are known reference standards (IC_50_ = 6 and 16 μg/mL, respectively) [21]. However, the obtained values are consistent with existing literature on oranges and orange peel studies. Specifically, the IC_50_ value (i.e., the sample concentration required to scavenge 50% of free radicals) of BO-pf was lower compared to ethanolic and methanolic extracts of orange peel [32,33], highlighting that BO-pf exhibits a non-negligible antioxidant activity. Notably, the total phenolic content of BO-pf (22.64 ± 0.20 mg GAE/g) is comparable to that of other citrus by-products, suggesting that the presence of neohesperidin (1.96 ± 0.06 mg/g) and naringin (0.88 ± 0.06 mg/g) may play a key role in its ability to neutralize reactive oxygen species (ROS) [26,34].

Several studies have investigated the biological activities of neohesperidin and naringin, which exhibit significant antioxidant, anti-inflammatory, cardioprotective, and antitumor properties by modulating oxidative stress, inflammatory pathways, and lipid and glucose metabolism [35,36].

Recent scientific observations have widely reported the health benefits of functional foods and natural bioactive compounds for the treatment and/or prevention of various chronic inflammatory diseases, including IBD.

Nutraceuticals, foods, or dietary supplements that provide concentrated forms of bioactive substances with medicinal properties are receiving considerable attention for the treatment of chronic diseases. The potential roles of functional foods against IBD have been extensively studied over the past decade, and overwhelming research evidence suggests that plant extracts, polyphenols, fatty acids, and amino acids can attenuate IBD symptoms by interfering with inflammatory pathways [37]. In this sense, a growing number of scientific studies are helping to clarify the molecular mechanisms underlying their beneficial action.

This study evidenced that BO-pf extract significantly reduced pro-inflammatory responses in intestinal epithelial cells exposed to the pro-inflammatory agent, LPS. In this regard, our data showed that, in LPS-stimulated HT29 and Caco2 cells, BO-pf extract pre-treatment significantly reduced the expression of IL-1β and IL-6 at the two concentrations tested.

Various cytokines and inflammatory mediators have been reported to play pivotal roles in IBD, including classical cytokines such as IL-1 and IL-6.

IL-1 includes a family of cytokines regulating the inflammatory response, including IL-1β. Macrophages mainly secrete this cytokine in response to the LPS of the bacterial wall. Still, it is also released by cells of the inflamed gastrointestinal mucosa in patients with IBD, triggering and exacerbating the intestinal inflammatory process [38,39].

IL-6 is also a pro-inflammatory cytokine with pleiotropic effects, which, together with IL-1β, initiates and intensifies the intestinal inflammatory process by amplifying the secretion of other inflammatory mediators (for example, IL-8 and eicosanoids). This mechanism leads to continuous and constant stimulation of the inflammatory process, which, becoming chronic, leads to tissue damage through the action of the proteolytic enzymes released in the damaged tissues and by the release of free oxygen radicals [40,41,42].

Conversely, IL-10 is a cytokine that has anti-inflammatory and protective effects. Consolidated experimental evidence highlights a link between the loss of IL-10 or its receptor and susceptibility to IBD [43,44]. Furthermore, it was demonstrated that mice deficient in IL-10 production developed chronic colitis. It has also been observed that IL-10 can reduce inflammation in both animal and in vitro models, confirming its role in reducing inflammation of the intestinal mucosa [45,46].

Similarly, transforming growth factor (TGF)β1 non-responsive mice and TGFβ1-null mice do not survive severe widespread autoimmunity, which also involves the intestine [47]. In the mammalian intestine, epithelial cells and numerous immune cells, particularly dendritic cells, produce TGFβ1, a regulatory cytokine of mucosal immune and inflammatory responses that contributes to maintaining intestinal homeostasis [48]. Elevated levels of TGFβ have been reported in patients with IBD, justifying the body’s attempt to regulate the extent of the inflammatory response. In this regard, it was demonstrated that serum TGFβ levels increased in response to conventional IBD treatments, suggesting that upregulation of TGFβ is necessary to attenuate intestinal inflammation in IBD patients [49,50].

Following robust local inflammation, the main consequence of the alteration of the intestinal barrier is the increase in mucosal permeability, with the entry of microbes, microbial toxins, and LPS from the intestinal lumen. This situation involves activating cells present locally, including dendritic cells, macrophages, and the epithelial cells themselves, with consequent sustained production of pro-inflammatory cytokines responsible for tissue inflammation and the interruption of homeostasis. TLR4 is an essential molecule in cellular activation by LPS, which leads to the activation of the NF-kB signaling pathway responsible for the transcription of genes related to the production of pro-inflammatory cytokines such as TNF-α, IL-1β, and IL-6 [51].

Clinical evidence suggests that well-planned dietary regimens with specific nutrients can alleviate gastrointestinal inflammation by modulating inflammatory cytokines, such as TNF-α, IL-1β, IL-6, and IL-10. In this context, fruit extracts, due to their potential anti-inflammatory and antioxidant properties, have been studied as therapeutic agents for treating IBD [52,53].

Polyphenols, typically abundant in fruits and vegetables, have been shown to modulate TLR and TLR4 signaling pathways, potentially providing anti-inflammatory benefits in IBD [54,55]. For example, curcumin, resveratrol, quercetin, and epigallocatechin gallate block TLR-mediated NF-kB activation, reducing inflammatory cytokine production [56,57].

Several studies have highlighted correlations between polyphenols present in citrus fruits, such as neohesperidin and naringin, and the modulation of pro- and anti-inflammatory interleukins. Orange peel and other citrus fruits are rich in bioactive compounds with antioxidant and anti-inflammatory properties, which can influence the immune response. Naringin and neohesperidin have been shown to reduce levels of IL-6, TNF-α, and IL-1β in experimental models of inflammation. Animal and cellular studies indicate that naringin can inhibit NF-κB, a key transcription factor involved in the production of pro-inflammatory cytokines [58,59]. Therefore, growing scientific evidence links the polyphenols in orange peel and other citrus fruits to regulating inflammatory interleukins, reducing inflammation and oxidative stress while promoting a more balanced immune response.

In line with previous observations reported in the literature, our results highlighted that BO-pf effectively reduced the expression of IL-1β and IL-6, with a pro-inflammatory action, and determined an upregulation of cytokines with anti-inflammatory action, such as IL-10 and TGFβ. Interestingly, we have further demonstrated that, in addition to the production of pro-inflammatory cytokines, BO-pf significantly suppresses the activation of pro-inflammatory signaling pathways, downregulating TLR4, p-IkBα, and NLRP3 inflammasome expression.

The NLRP3 inflammasome has emerged as a crucial regulator of intestinal homeostasis and has been widely associated with the pathogenesis and progression of IBD [60,61]. Several experimental observations highlight that suppression of NLRP3 is effective in alleviating IBD [62].

Inflammasomes are cytosolic multiprotein oligomers of the innate immune system that activate inflammatory responses [63]. It is a critical component of the innate immune system that mediates caspase-1 activation and the secretion of pro-inflammatory cytokines IL-1β/IL-18 in response to microbial infection and cellular damage, enhancing inflammation in the gut and aggravating colonic damage [64].

In our LPS-stimulated cells, BO-pf treatment significantly reduced caspase-1 expression and IL-1β expression, highlighting that the extracts could inhibit NLRP3 inflammasome-induced caspase-1 cleavage and subsequent production of IL-1β. In this regard, studies have reported that suppressing NLRP3 activation led to the inhibition of pro-inflammatory cytokine release, reducing anti-inflammatory responses in macrophages, underlying the therapeutic regulation of the NLRP3 inflammasome in chronic inflammatory diseases [65,66,67].

At the same time, we observed a slight but significant increase in NLRP3 expression in both intestinal cell types in the presence of BO-pf extracts alone.

A mild, transient increase in inflammasome activation induced by polyphenols in intestinal cells presents a fascinating paradox. While inflammasomes are typically associated with pro-inflammatory responses and disease, emerging research suggests that a subtle, controlled activation can confer protective effects. In this respect, in a model of *Citrobacter rodentium* infection, it was demonstrated that mice lacking NLRP3 and caspase-1 showed increased susceptibility to bacterial penetration at the level of the intestinal crypts [68]. Furthermore, mutations in the downstream regulatory region of NLRP3 have also been shown to be associated with decreased IL-1β expression and increased susceptibility to Crohn’s disease (CD) in humans [69]. Indeed, it has been observed that IL-1β also plays a fundamental role in intestinal epithelial repair and the formation of the epithelial barrier in a dextran sodium sulfate (DSS) colitis model, since it has been observed that IL-1β deficiency reduces intestinal epithelial cell proliferation and the expression of tight junction proteins compromises intestinal permeability. Impaired epithelial barrier function in NLRP3-deficient mice was evidenced by increased translocation of commensal bacteria into colonic tissue and subsequently increased dissemination to other peripheral organs [70]. Finally, NLRP3-deficient mice were reported to be susceptible to colitis, and this condition was, at least in part, determined by increased epithelial barrier damage. It is known that epithelial damage can induce a localized repair response by increasing the division of stem cells present at the base of crypts to replace damaged enterocytes [71,72]. In this respect, it was demonstrated that, in NLRP3-deficient mice, the proliferation rate of epithelial cells in the colon during acute DSS colitis was significantly reduced. These results led to the postulate that NLRP3 seems to have a crucial protective role in maintaining the epithelial barrier by promoting the proliferation of epithelial stem cells [73].

Only a few inflammasomes can regulate intestinal homeostasis and inflammation; among these is NLRP3 [74]. Therefore, the NLRP3 activation in intestinal epithelial cells seems linked, at least partially, to an essential physiological role in maintaining the intestinal barrier, limiting pathogen colonization [75].

Moreover, it is also known that, besides infectious diseases, NLRP3 activation plays a central role in autoimmune and inflammatory diseases. In this regard, the ability of NLRP3 to protect against the development of colorectal cancer is attributed to the effector function of caspase-1 to mediate the secretion of IL-18. This key cytokine promotes epithelial barrier regeneration during the early stages of colitis [76].

These observations are particularly relevant in the context of dietary polyphenols. Accordingly, a low-level modulated activation of inflammasomes could play a role in cellular homeostasis and defense. Currently, there is no evidence in the literature on the ability of polyphenols to determine a controlled modulation of the activation of inflammasomes, including NLRP3. Based on our experimental results, it can be speculated that a slight increase in inflammasome activation determined by the polyphenols contained in the BO-pf extracts may have a protective effect on intestinal cells, both in physiological conditions and in the presence of an inflammatory state. This possibility can be hypothesized with regard to both the polyphenols present in the BO-pf extracts used in our work and other polyphenols. This leads to a challenge to the traditional view of inflammasomes as exclusively pro-inflammatory agents. This intriguing emerging perspective again highlights the complex interaction between diet, inflammation, and intestinal homeostasis. Further research is certainly needed to study in more detail the mechanisms of NLRP3 modulation by polyphenols, potentially laying the foundations for possible new therapeutic strategies to promote correct intestinal homeostasis.

Therefore, by targeting these signaling pathways, nutraceutical active substances, such as polyphenols, can suppress the activation of immune cells, reduce cytokine production, and promote tissue repair. Clinically, this could lead to decreased disease activity, fewer flare-ups, and improved mucosal repair, thus enhancing the quality of life of patients with inflammatory bowel disease.

## 5. Conclusions

The main results of our study highlight that citrus waste and by-products, such as BO-pf, represent potential bioactive compounds with notable biological activities. Specifically, BO-pf extract is a rich source of polyphenolic compounds with antioxidant properties. Furthermore, as demonstrated by our in vitro results, in LPS-activated intestinal epithelial cells, BO-pf treatment significantly reduced the protein expression of NLRP3, TLR4, and p-IkBα and significantly inhibited caspase-1 cleaved levels. In addition, BO-pf treatment determined suppression of pro-inflammatory cytokine production, in terms of IL-1β and IL-6 expression, via inhibition of NF-kB and NLRP3 inflammasome signaling cascades. Finally, BO-pf treatment significantly upregulated the expression of the anti-inflammatory cytokines with protective activity, namely IL-10 and TGFβ, thus suggesting a potential role for BO-pf in the treatment and/or prevention of IBD.

## Figures and Tables

**Table 1:** Chemical composition, antioxidant activity, and polyphenol content of BO-pf.

Parameters	BO-pf
Moisture (g/100 g)	8.14 ± 0.05
pH	4.15 ± 0.01
Protein (g/100 g)	5.08 ± 0.05
Lipid (g/100 g)	1.12 ± 0.04
Fiber (g/100 g)	35.2 ± 0.08
Carbohydrates (g/100 g)	44.7 ± 0.35
Salt (g/100 g)	˂0.1
ABTS (µmol TE/g)	21.94 ± 0.59
DPPH (µmol TE/g)	20.87 ± 0.40
IC_50_ (μg/mL)	725.00 ± 7.07
TPC (mg GAE/g)	22.64 ± 0.20
Neohesperidin (mg/g)	1.96 ± 0.06
Naringin (mg/g)	0.88 ± 0.06

Data are represented as means ± SD of three lots of BO-pf. Abbreviations: ABTS, 2,2′-azino-bis (3-ethylbenzothiazoline-6-sulfonic acid); BO-pf, blood orange peel flour; DPPH, 2,2-diphenil-1-picrylhydrazyl; TPC, total phenolic content.

## Data Availability

The original contributions presented in this study are included in the article. Further inquiries can be directed to the corresponding author.
